# Supplementary material for: Movement Sonification Techniques to Improve Balance in Parkinson’s Disease: A Pilot Randomized Controlled Trial
Source: Brain Sci. 2023 Nov 12;13(11):1586. doi: 10.3390/brainsci13111586 (PMC10670131; doi:10.3390/brainsci13111586)

Figure S11: SonicWalk Operational Framework

SONICWALK

OPERATIONAL FRAMEWORK

SENSORS CALIBRATION

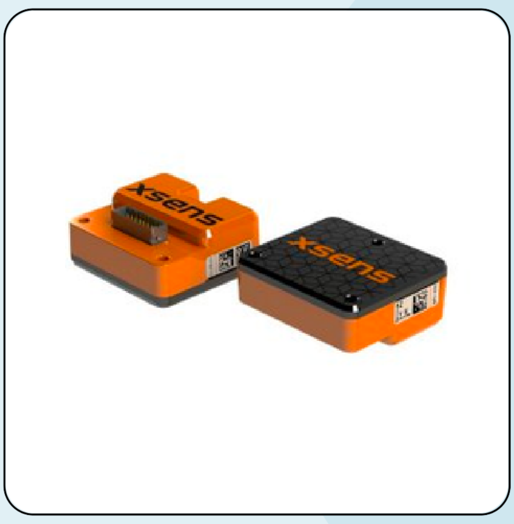

2 Xsens inertial sensors, positioned one per leg at the ankle (Xsens Technologies B.V., The Netherlandsthe)

Cadence is calculated during the initial phase of sensors calibration, where the patient is asked to walk normally.

MOTION DATA ANALYSIS

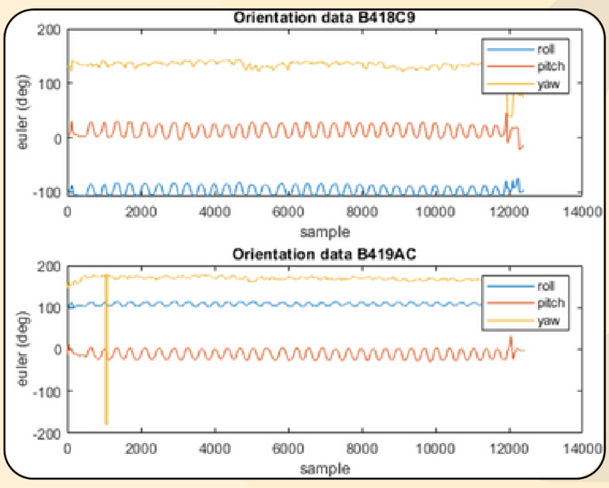

Motion Data Retrieved from the sensors: instantaneous angular position detection related to the knee joint.

DATA PROCESSING

Processing:

1) Foot strike detection, step cadence calculation.

2) Association of sounds with movements (sonification) using a specific algorithm.

SONIFICATION EXAMPLE

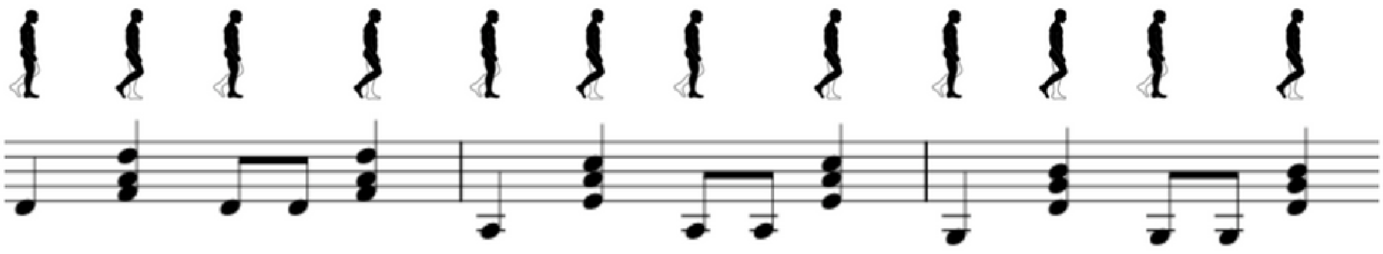

Supplement: Supplementary file 1 [file brainsci-13-01586-s001.zip › Figure S11 SonicWalk Operational Framework.pdf]
