# Supplementary material for: Movement Sonification Techniques to Improve Balance in Parkinson’s Disease: A Pilot Randomized Controlled Trial
Source: Brain Sci. 2023 Nov 12;13(11):1586. doi: 10.3390/brainsci13111586 (PMC10670131; doi:10.3390/brainsci13111586)
Supplement: Supplementary file 1 [file brainsci-13-01586-s001.zip › Figure S1_SonicWalk Protocol.pdf]

Figure S1: SonicWalk Protocol

SONICWALK
PROTOCOL

20 SESSIONS // 30' EACH // 3 TIMES/WEEK

ASSESSMENT: BASELINE (PRE), END OF TREATMENT (POST), FOLLOW-UP (FU).

CADENCE ASSESSMENT

The baseline pace of the patient is measured. The patient is asked to walk at his/her comfortable speed (the measure is obtained considering a click every half step)

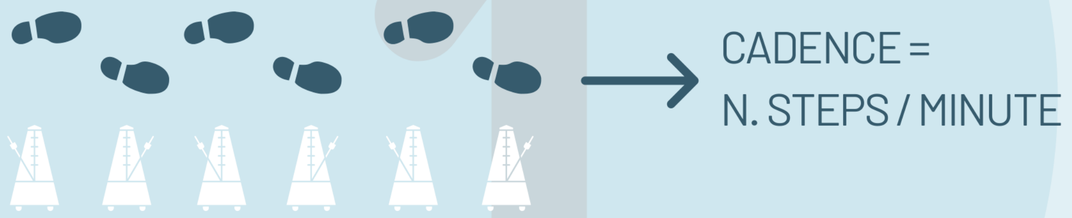

STANDARD GAIT REHABILITATION	GAIT WITH SONIFICATION

15' WARM-UP PHASE

STANDARD GAIT REHABILITATION

3 MINUTES EACH, WITH A SHORT BREAK IN THE MIDDLE

1. Anteroposterior load shift in tandem position, left foot forward
2. Anteroposterior load shift in tandem position, right foot forward
3. Left foot swing
4. Right foot swing
5. March in place

GAIT WITH SONIFICATION

SAME EXERCISES BUT WITH MUSICAL CUES

- **1':30"** an external musical cue entrains the gait
- **1':30" Sonification Phase**: same musical patterns are re-created naturally, in real-time, by patient walk (without metronome)

15' GAIT PHASE

7' WALK

Standard: The patient performs a **7-minute walk**

Sonification: The patient performs a **7-minute walk** guided by a chord progression

1' BREAK

Standard: **1 minute** break

Sonification: **1 minute** break

7' WALK WITH INCREASING PACE

Standard: The patient performs a **7-minute walk** slightly increasing the gait pace to the maximum possible speed

Sonification: **Sonification phase**: The patient reproduces the chord progression in real-time, without metronome, associating each foot contact with a chord. The patient, along the **7-minute walking**, is asked to slightly increase the pace of their steps to the maximum possible speed
