# Supplementary material for: Movement Sonification Techniques to Improve Balance in Parkinson’s Disease: A Pilot Randomized Controlled Trial
Source: Brain Sci. 2023 Nov 12;13(11):1586. doi: 10.3390/brainsci13111586 (PMC10670131; doi:10.3390/brainsci13111586)

Figure S2: Scheme of training with music

SCHEME OF TRAINING WITH MUSIC

TOTAL TIME	PHASE	SUB PHASE	SUB PHASE TIME	KIND OF SONIFICATION
15′	**WARM-UP 1** Anteroposterior load shift in tandem position, left foot forward	W1A	1′:30″	PRE-RECORDED STIMULI + METRONOME
		W1B	1′:30″	REAL-TIME SONIFICATION
	**WARM-UP 2** Anteroposterior load shift in tandem position, right foot forward	W2A	1′:30″	PRE-RECORDED STIMULI + METRONOME
		W2B	1′:30″	REAL-TIME SONIFICATION
	**WARM-UP 3** Left foot swing	W3A	1′:30″	PRE-RECORDED STIMULI + METRONOME
		W3B	1′:30″	REAL-TIME SONIFICATION
	**WARM-UP 4** Right foot swing	W4A	1′:30″	PRE-RECORDED STIMULI + METRONOME
		W4B	1′:30″	REAL-TIME SONIFICATION
	**WARM-UP 5** March in place	W5A	1′:30″	PRE-RECORDED STIMULI + METRONOME
		W5B	1′:30″	REAL-TIME SONIFICATION
15′	**GAIT**	GA	7′	PRE-RECORDED STIMULI + METRONOME
		REST	1′	-
		GB	7′	REAL-TIME SONIFICATION

Figure S3: warm-up 1A and 2A

W1A, W2A - PRE-RECORDED STIMULI - ⏱ 1′:30″ + ⏱ 1′:30″

Anteroposterior load shift in tandem position, RIGHT / LEFT foot forward

A metronome and an external musical cue entrain the movement

BEAT	1		2		3		4	
**METRONOME**<br><br>BPM = patients' spontaneous cadence (metronome is played independently)	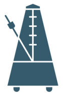		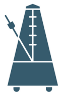		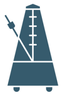		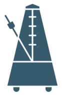	
**MUSICAL CUE: CHORD PROGRESSION EXAMPLE**	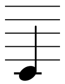		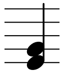		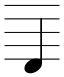		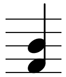	
**RIGHT FOOT**	Toe tip up		Foot placement		Toe tip up		Foot placement	
	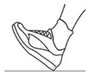	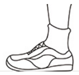	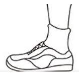	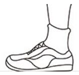	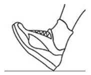	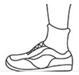	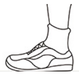	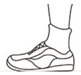
**LEFT FOOT**	Foot placement		Heel up		Foot placement		Heel up	
	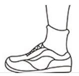	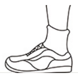	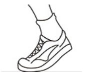	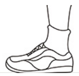	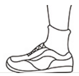	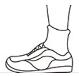	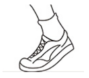	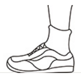
**MOVEMENT EXAMPLE**	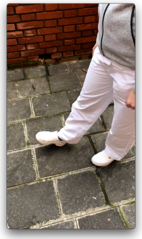	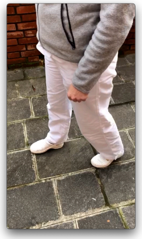	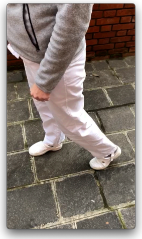	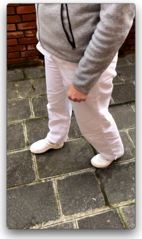	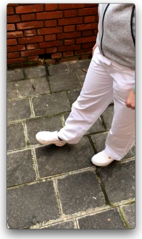	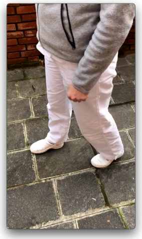	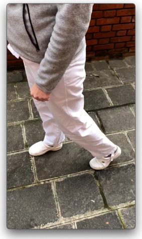	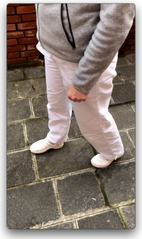

Figure S4: warm-up 1B and 2B

W1B, W2B - REAL-TIME SONIFICATION - ⏱ 1′:30″ + ⏱ 1′:30″

Anteroposterior load shift in tandem position, RIGHT / LEFT foot forward

Sonification Phase: same musical patterns are re-created naturally, in real-time, by patient movement (without metronome)

**REAL-TIME SONIFICATION**<br>CHORD PROGRESSION EXAMPLE<br>Footstep = single progression fragment	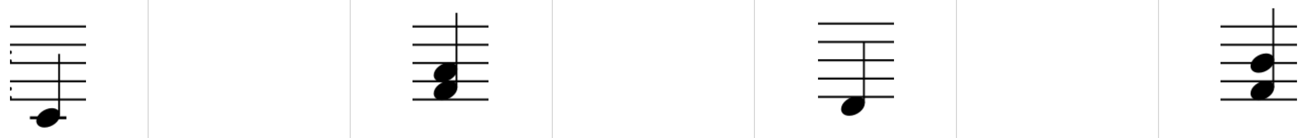							
**RIGHT FOOT**	Toe tip up		Foot placement		Toe tip up		Foot placement	
	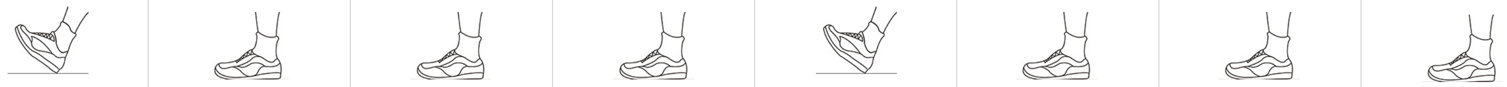							
**LEFT FOOT**	Foot placement		Heel up		Foot placement		Heel up	
	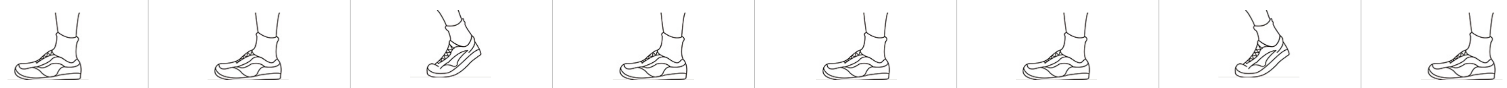							
**MOVEMENT EXAMPLE**	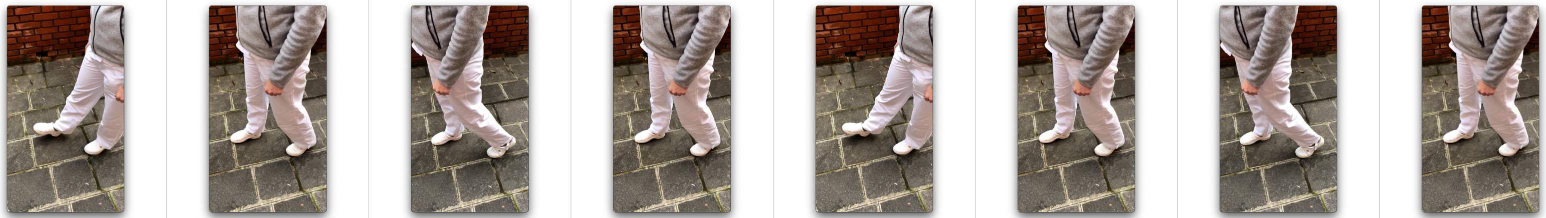							
**COMPLETE MUSICAL SEQUENCE**	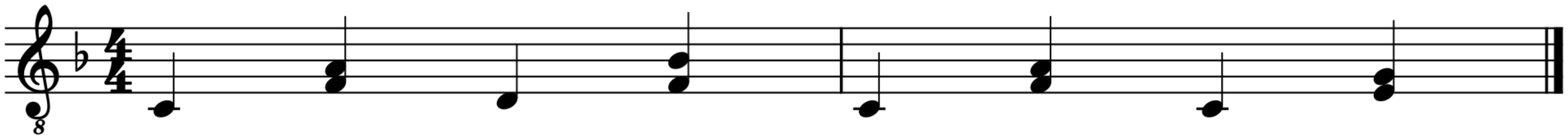							

Figure S5: warm-up 3A and 4A

W3A, W4A - PRE-RECORDED STIMULI - ⏱️ 1':30" + ⏱️ 1':30"

Left / Right foot swing

A metronome and an external musical cue entrain the movement

**BEAT**	1	2	3	4	1	2	3	4
**METRONOME**<br><br>BPM = patients' spontaneous cadence (metronome is played independently)								
**MUSICAL CUE: CHORD PROGRESSION EXAMPLE**	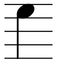	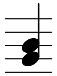	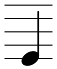	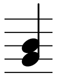	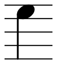	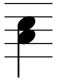	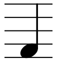	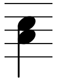
**MOVEMENT EXAMPLE**	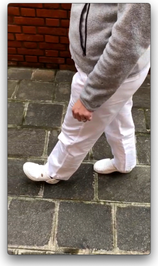	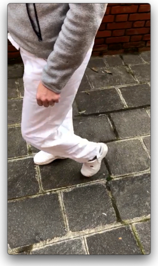	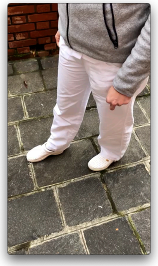	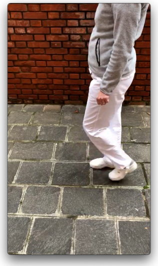	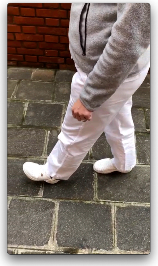	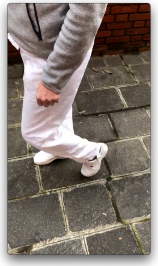	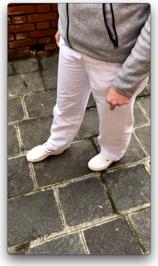	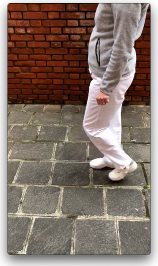
**LEFT SWING**	Left foot forward placement	Left swing (the left foot moves backward)	Left foot backward placement	Left swing (the left foot moves towards)	Left foot forward placement	Left swing (the left foot moves backward)	Left foot backward placement	Left swing (the left foot moves towards)

Figure S6: warm-up 3B and 4B

W3B, W4B - REAL-TIME SONIFICATION - ⏱ 1':30" + ⏱ 1':30"

Left / Right foot swing

Sonification Phase: same musical patterns are re-created naturally, in real-time, by patient movement (without metronome)

**REAL-TIME SONIFICATION**<br>CHORD PROGRESSION EXAMPLE<br>Footstep and intermediate swing phase = single progression fragment	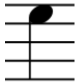	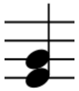	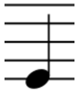	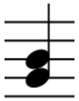	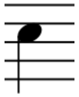	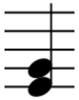	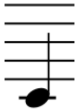	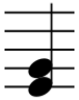
**MOVEMENT EXAMPLE**	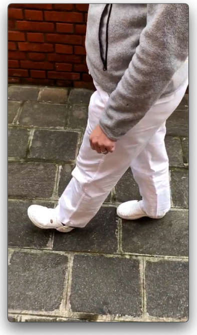	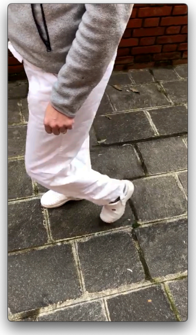	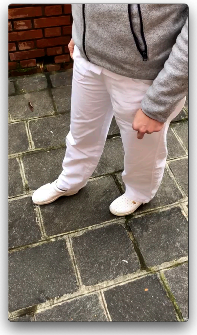	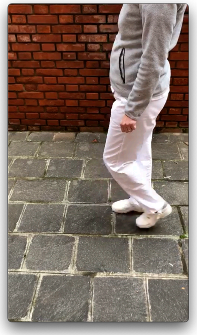	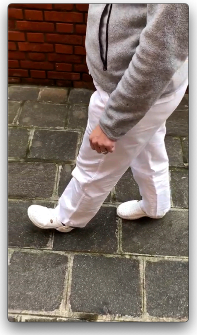	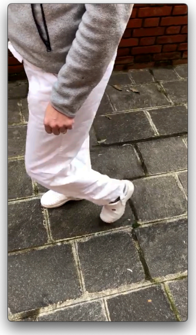	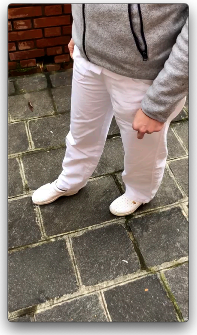	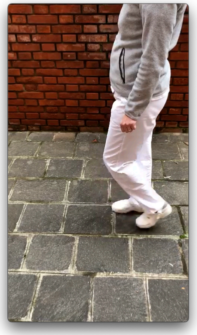
**LEFT SWING**	Left foot forward placement	Left swing (the left foot moves backward)	Left foot backward placement	Left swing (the left foot moves towards)	Left foot forward placement	Left swing (the left foot moves backward)	Left foot backward placement	Left swing (the left foot moves towards)
**COMPLETE MUSICAL SEQUENCE**	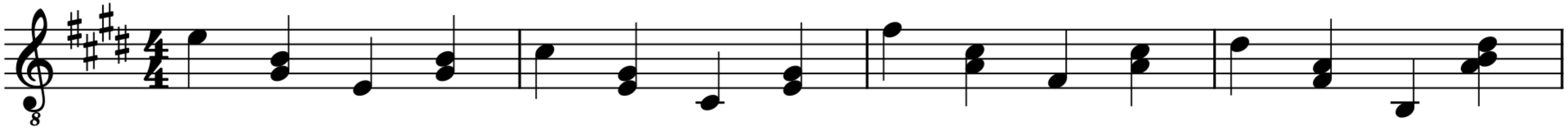							

Figure S7: warm-up 5A

W5A - PRE-RECORDED STIMULI - ⏱ 1′:30″

March in place

A metronome and an external musical cue entrain the movement

**BEAT**		1		2		3	^	4		1		2		3	^	4	
**METRONOME**<br>BPM = patients’ spontaneous cadence (metronome is played independently)																	
**MUSICAL CUE: CHORD PROGRESSION EXAMPLE**																	
**RIGHT FOOT**																	
**LEFT FOOT**																	

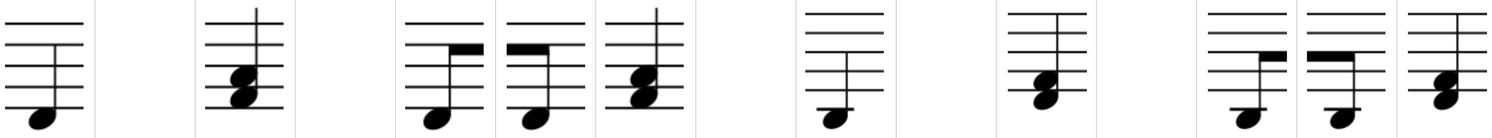

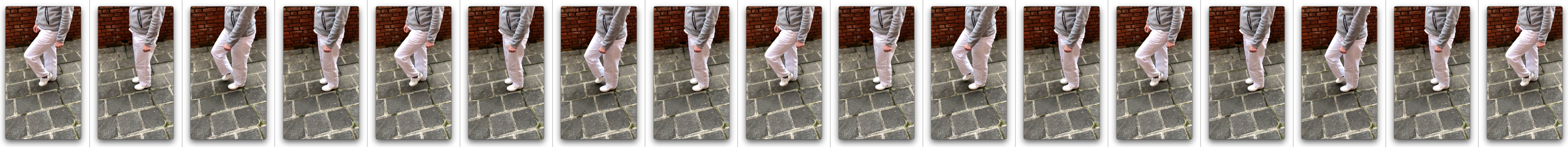

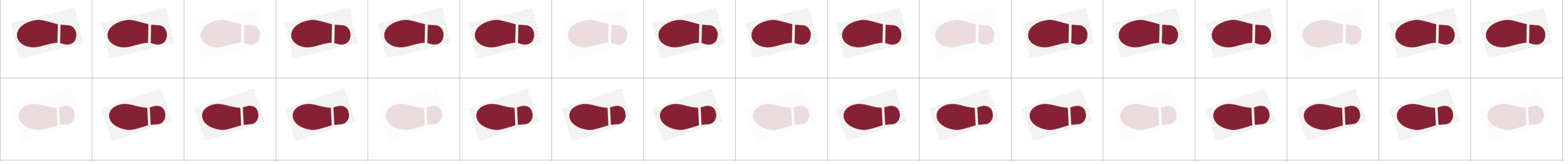

Figure S8: warm-up 5B

W5B - REAL-TIME SONIFICATION - ⏱ 1’:30”

March in place

Sonification Phase: same musical patterns are re-created naturally, in real-time, by patient movement (without metronome)

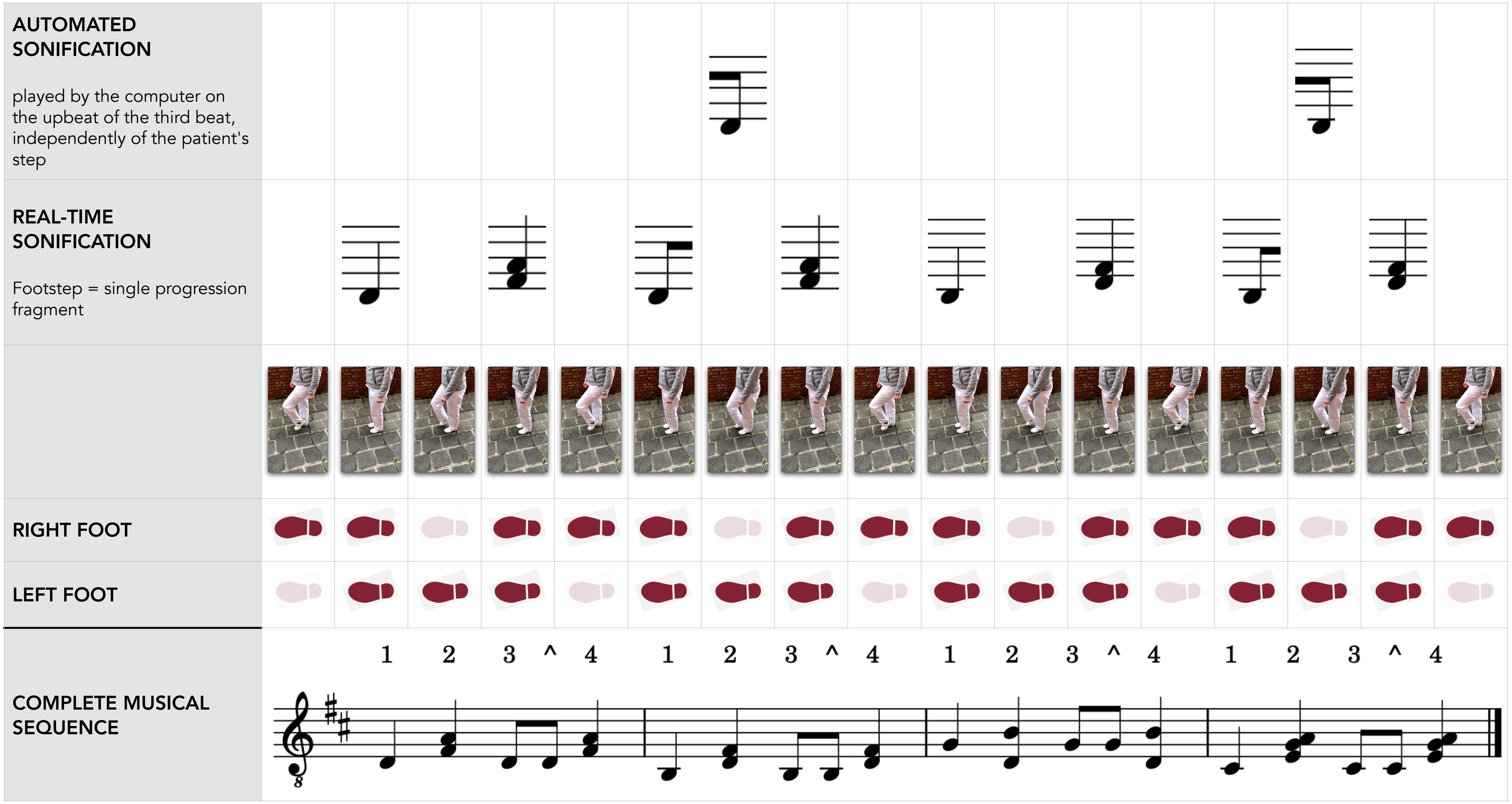

Figure S9: Gait A

GA - PRE-RECORDED STIMULI - ⏱ 7’

Gait

A metronome and an external musical cue entrain the gait

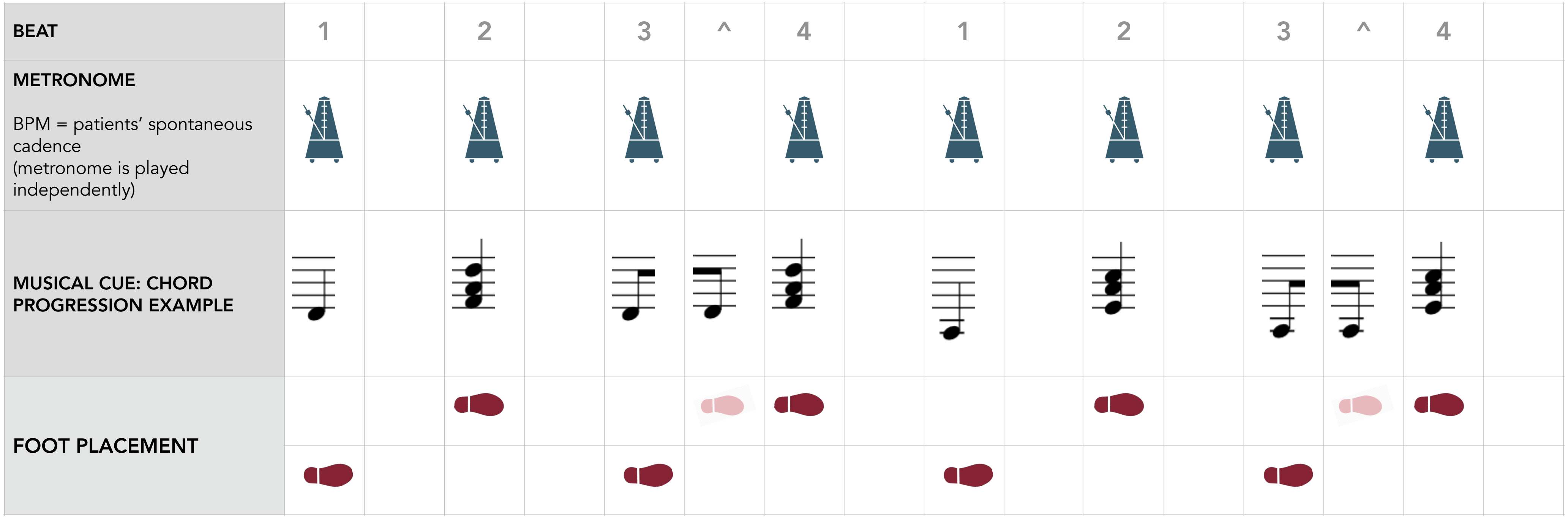

Figure S10: Gait B

GB - REAL-TIME SONIFICATION - ⏱️ 7’

Gait

Sonification Phase: same musical patterns are re-created naturally, in real-time, by patient walk (without metronome). The patient is asked to gradually increase the pace to the maximum possible speed.

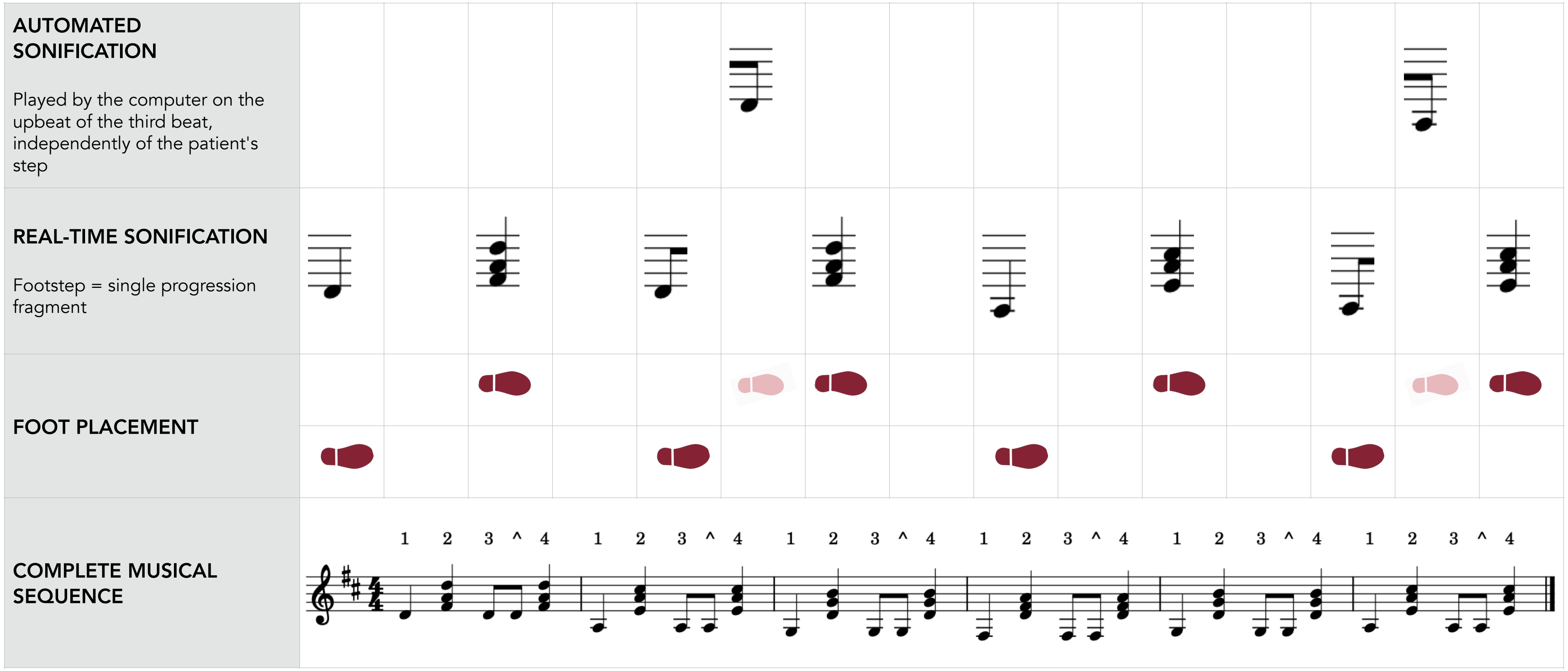

Supplement: Supplementary file 1 [file brainsci-13-01586-s001.zip › Figures S2_S10_Scheme of training with music .pdf]
